# Encapsulation of Carbon Dots in a Core–Shell Mesh through Coaxial Direct Ink Writing for Improved Crop Growth

**DOI:** 10.1021/acssuschemeng.3c02641

**Published:** 2023-09-11

**Authors:** Isik Arel, Ayse Ay, Jingyi Wang, Luz Karime Gil-Herrera, Ahu Gümrah Dumanli, Ozge Akbulut

**Affiliations:** † Faculty of Engineering and Natural Sciences, Sabanci University, Tuzla, Istanbul 34956, Turkey; ‡ Department of Materials, University of Manchester, Manchester M13 9PL, U.K.; § Henry Royce Institute, The University of Manchester, Oxford Road, Manchester M13 9PL, U.K.

**Keywords:** carbon dots, controlled release, core−shell fabrication, direct ink writing, coaxial printing, nanofertilizer, crop growth

## Abstract

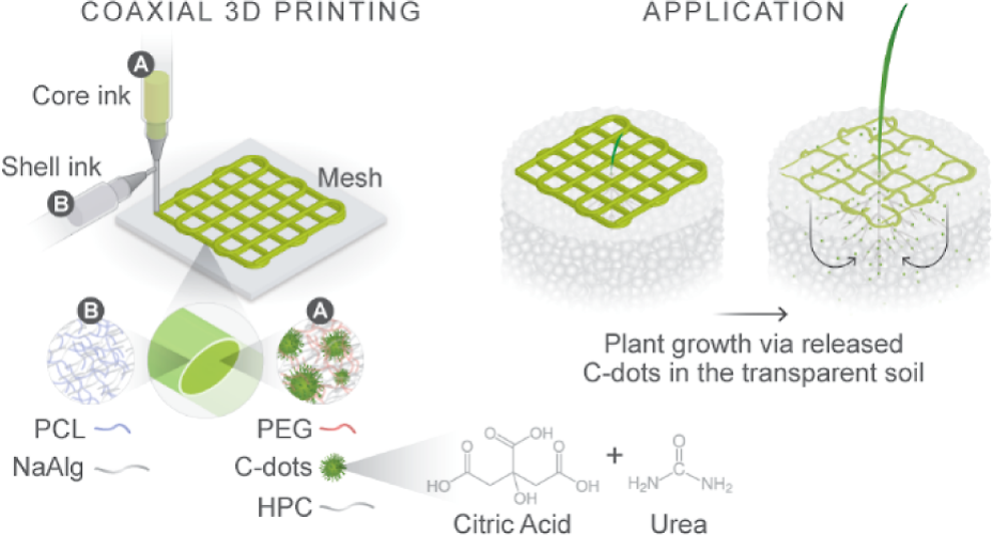

Through coaxial direct ink writing, we fabricated a core–shell mesh system for the controlled release of carbon dots (C-dots). In the core ink, we developed an ink formulation with tuned viscosity using hydroxypropyl cellulose and polyethylene glycol to host C-dots. Polycaprolactone was employed as the main shell material, in combination with sodium alginate, to control the degradation rate of the shell. We investigated the degradation profile of the 3D-printed meshes and tracked the weekly release of C-dots in an aqueous medium by spectrofluorometry. We tested the efficacy of the C-dot release on plants by placing the meshes in transparent soil with *Triticum aestivum* L. seeds. We observed the in vivo translocation of the C-dots in the plant using confocal microscopy. We measured the root elongation and shoot length to assess the effect of C-dots on plant growth. Our study revealed that the plants exposed to C-dots grew 2.5-fold faster than the control group, indicating that C-dots are promising nanofertilizers for aggrotech and non-toxic fluorescent biolabels for in vivo applications.

## Abstract 2

We fabricated core−shell meshes of C-dots through direct ink writing as a controlled release system for agricultural applications.

## Introduction

Carbon dots (C-dots) are a new generation of fluorescent particles that can offer biocompatibility, tunable photoluminescence, water solubility, high photobleaching, and photoblinking resistance.^1^ Fluorescence quantum yields and solubility of C-dots can be easily enhanced by modifying their surface functional groups.^2,3^ This ease of chemical functionality and inertness of C-dots make them desirable for several fields such as diagnostics,^4,5^ pharmacology,^6,7^ and bio-tracking.^8^

The zero-dimensionality of C-dots enables ease in absorption and penetration through the cell wall, and their fluorescent behavior provides the ability to track their dispersion.^9−12^ Recently, even at trace amounts, C-dots have been shown to promote plant growth by increasing light harvesting efficiency and, in turn, photosynthesis.^13−15^ Such photosynthetic efficiency of C-dots has been attributed to their ability to down-convert the UV light to blue and red light to match the absorption spectrum of chloroplasts precisely. In these proof-of-concept studies, C-dots were applied to the crop growth media through the nutrient solution,^13^ seed priming,^14^ root exposure,^15^ or spraying on the seeds.^21^ To be able to harness the desired properties of C-dots in agricultural settings, a scalable, biocompatible, and cost-effective delivery method should be devised. In such a timely manner, there is an ongoing shift in the agricultural industry toward controlled release systems to prevent the excessive use of agrochemicals (e.g., fertilizers or pesticides).^22,23^ The controlled release approach enables a long residence of these chemicals in the soil, prolongs their shelf life, and offers ease of transport. Currently, most of the controlled release systems are in the particle form, and their design relies on the encapsulation of a chemical of interest—core—with a degradable polymer shell. This shell allows the delivery of the core over a period of time and protects the core against environmental factors. However, the chemical fabrication routes to produce these systems require high-cost precursors, organic solvents, high temperatures, or a vacuum environment, thus generating a high carbon footprint. Additionally, achieving a consistent shell thickness and homogeneity has been shown to be problematic.^24^ Compared to the particulate systems, core–shell structures in the fiber form can contribute to the homogeneous distribution of active ingredients.^25^ Electrospinning, so far, has been a straightforward, room-temperature method to envelope fertilizers, pesticides, and herbicides in this form.^26,27^ Nevertheless, electrospinning also utilizes excessive amounts of organic solvents, and the fabrication of fibers with proper mechanical strength is still challenging due to the limitation of the fiber diameter that can be produced via this method. The micron-sized fibers can only encapsulate a scarce amount of material; therefore, the reports on the applications of electrospun fibers in agriculture only cover short-term release studies (e.g., hours to a couple of days).^28,29^

Coaxial direct ink writing (DIW) offers a room-temperature process via co-extrusion of the core and shell inks. This mechanical route generates minimum waste and produces robust fibers in the sub-mm/mm range with loadings that are relevant to agricultural applications. In this work, through DIW, we printed meshes of core–shell fibers that encapsulated C-dots in a degradable shell. We focused on polycaprolactone (PCL) for the shell formulation due to its biodegradability, mechanical strength, and flexibility. Since the slow degradation of PCL can pose a challenge for controlled release applications,^30−32^ we tuned its degradation rate by blending it with sodium alginate (NaAlg).^33^ For the core ink to host C-dots and ensure compatibility with the shell ink, we systematically explored two viscosity modifiers, hydroxypropyl cellulose (HPC) and polyethylene glycol (PEG). We tracked the degradation profile of the printed meshes by measuring the electrical conductivity arising from Na^+^ ions upon degradation of the PCL/NaAlg shell. We analyzed the time-dependent delivery pattern of C-dots based on their emission spectra by spectrofluorometry. The in vivo transport route of C-dots from the root to the stem through the vascular system in a model plant (*Triticum aestivum* L.) was evaluated by confocal microscopy. We visually investigated and measured the root and shoot growth in lab-made transparent soil. Our work showed that the seeds exposed to C-dots displayed 2.5-fold faster growth than the plant without any treatment. We demonstrated the robustness of the meshes for storage and transportation by assessing their foldability and ability to carry loads through mechanical characterization. Our work suggests an effective and sustainable delivery method to store, carry, and deliver C-dots in a stable environment. Moreover, tracking the uptake and translocation of C-dots through the plant tissues confirmed their potential as highly effective tools for in vivo bioimaging. This benchtop, room-temperature prototyping method presented here is scalable and eco-friendly for the fabrication of functional, robust, and foldable agricultural textiles for controlled delivery applications.

## Experimental Section

### Materials

PEG (molecular weight, *M*_w_, of 200, 600, and 1000 g/mol) was purchased from Merck. HPC (*M*_w_ 100,000 g/mol) was obtained from Alfa-Aesar. NaAlg, chloroform, PCL pellets (*M*_w_ ∼ 45,000), citric acid, urea, phytagel, magnesium chloride hexahydrate (MgCl_2_), Murashige and Skoog basal medium, and Pluronic F127 were purchased from Sigma-Aldrich. Calcium chloride dihydrate (CaCl_2_, food grade) was obtained from ZAG Kimya. All chemicals and reagents were used without purification. *T. aestivum* L. seed (product no 1016) was purchased from Tekfen Tarım. Deionized water was purified using a MilliQ water purification system (conductivity = 18.2 Ω).

### Synthesis and Characterization of C-Dots

A facile one-step method was used to prepare C-dots by modifying the protocol developed by Vercelli.^34^ In summary, 0.2 M citric acid and 0.6 M urea were dissolved in 20 mL of deionized water to form a clear solution under magnetic stirring for 5 min. The mixture was transferred to a sealed Teflon-lined stainless steel autoclave and kept at 160 °C for 4 h. The reaction was finalized by cooling the autoclave vessel at room temperature, and the reactant solution was then centrifuged at 10,000 rpm for 10 min to remove the insoluble solid precipitate. To achieve purification of the C-dots, the hydrothermally produced C-dot suspension was dialyzed using dialysis tubing (Spectra/Por) with a 2 kDa molecular weight cut-off for 48 h. This method effectively reduces the presence of impurities below the molecular weight cut-off, including ions and small byproducts, while retaining the synthesized C-dots within the tubing, thereby guaranteeing proper purification. The dispersed C-dots in the water were then collected and freeze-dried.

Transmission electron microscopy (Talos F200X G2, operated at 200 kV, Thermo Fisher, USA) was used to analyze the morphology and size distribution of the as-prepared C-dots. Image acquisition was carried out with Velox software. The TEM image analysis was performed using ImageJ (National Institutes of Health, Wayne Rasband, Bethesda, MD, USA). The particle size and size distribution were determined by averaging more than 50 particles. The particle size of the C-dots was in the range of 10–50 nm (Figure S1). The Gaussian fitting curves demonstrated that the average diameter of the corresponding products was 22.5 nm (Figure S1). The UV–vis spectra were recorded on a Lambda 365 (PerkinElmer, USA) in the 200–700 nm wavelength range. The fluorescence intensity measurements of C-dots were conducted at room temperature on a fluorimeter (Edinburgh Instruments FL900, UK). The emission spectrum was obtained by exciting the C-dots solution of 0.05 mg/mL at 360, 380, and 400 nm using a slit width of 5 mm for outgoing and incoming beams and measuring emission up to 700 nm. Raman spectroscopy was performed on a LabRAM HR Evolution instrument (Horiba Scientific, Japan) equipped with a He–Ne laser (wavelength 633 nm) with an edge filter and a 600 g/mm grid. Prior to the spectral analysis, the C-dot suspensions were cast and flattened on a clean silicon wafer, and a 50× long working distance objective was used to focus the laser on the samples with a laser power of 0.29 mW.

### Preparation of the Inks for 3D Printing

For the shell inks, we explored formulations that contain a PCL range of 13–20 wt % with 1.5–8 wt % NaAlg addition. The optimized formulation of 14.32 wt % PCL and 5.73 wt % NaAlg, corresponding to a PCL-to-NaAlg ratio of 2.5:1, was prepared by dissolving 2.5 g of PCL pellets and 1 g of NaAlg powder in 5 mL of chloroform and 6.5 mL of deionized water, respectively. These two solutions were mixed and magnetically stirred (∼300 rpm) for 5 h at room temperature. For the first core ink, we fixed the amount of C-dots to 0.1 mg in 1 mL of deionized water and analyzed the addition of PEG200 (3–20 wt %) and HPC (5–35 wt %) to this aqueous suspension. The optimum formulation contained 0.071 g (5 wt %) of PEG200. The PEG–C-dot mixture was slowly stirred (∼300 rpm) for 1 h, and 0.36 g (25 wt %) of HPC was introduced afterward, and the stirring continued for another 7 h until an entirely homogeneous mixture was obtained. For the second core ink, we dissolved 0.1 mg of C-dots in 1 mL of deionized water and added HPC in the range of 5–35 wt %. Based on the printing performance, the optimum amount of HPC was 0.43 g, corresponding to an addition of 25 wt %. This mixture was kept under magnetic stirring for 8 h at room temperature.

### Rheological Characterization of the Inks

The rheological analysis of the inks was carried out using a rheometer with a cone plate geometry of 25 mm/2° and a fixed gap size of 0.103 mm (Anton-Paar MCR302, Austria). In a dynamic regime, we set the frequency to 10 rad s^–1^ and changed the strain from 0.01 to 1000% to find the range of the linear viscoelastic region. In the steady-state tests, the shear rate ranged from 0.01 to 1000 s^–1^.

### 3D Printing of the Inks

All inks were printed via a pneumatic bioprinter (Axolotl A1 bioprinter, Axolotl Biosystems, Türkiye) in a coaxial configuration onto a Teflon surface to ease the removal of meshes at room temperature. The inks were gently mixed and loaded into 3 cm^3^ print cartridges equipped with a 15/19-gauge stainless steel metal coaxial nozzle. Prior to the printing, Repetier-Host (Hot-World GmbH & Co. KG, Willich, Germany) and Slic3r as a slicing engine were used to convert the pre-prepared STL files into the geometry-code (G-code) file. The grid infill pattern was selected to generate a mesh structure. The printing head moved in the *X* and *Y* directions, and the stage moved in the *Z* direction. The printing speed was kept constant at 1 mm/s, the infill percentage was 8%, and the layer height was 0.2 mm. The distance between the Teflon surface and the nozzle tip was fixed at approximately 0.1 mm to prevent potential sagging and buckling due to the gravitational force.

### Imaging

We analyzed the core–shell structure of the 3D-printed meshes through field emission scanning electron microscopy (SEM, Zeiss Leo Supra 35VP, Germany). After drying, the printed samples were physically fractured to visualize the core–shell structure. The samples were fixed onto specimen stubs with a 90° angle to capture the cross-sectional fracture surface. To prevent charging, the samples were coated (∼9 nm) with Au/Pd by using a Cressington 108 sputter coater at a current of 40 mA for 30 s. The core and shell thickness of the printed meshes were estimated using ImageJ. The 3D-printed meshes were photographed by a Spot Insight QE camera (Diagnostic Instruments, Silver Spring, USA) at 5× and 10× magnifications in optical microscopy (Nikon Eclipse ME600, Japan) (Figure S2). We investigated the translocation and uptake of C-dots via confocal microscopy (Carl-Zeiss LSM 710, Zeiss AXIO Observer Z1, Germany) with 10×/03 and 20×/0.8 M27 dry objectives. Images were captured by using Zen imaging software. The plants were exposed to C-dots for a duration of 15 days. On day 16, the plants were carefully harvested and dissected into their respective tissue parts (stem, lateral root, and taproot), which were then placed in a confocal Petri dish and sequentially excited at 405, 488, and 561 nm to validate whether C-dots are capable of accumulating within the tissues of plants compared to the control group.

### Degradation Kinetics of the PCL/NaAlg Shell

The hydrolytic degradation profile of the PCL/NaAlg shells was obtained by immersing the PCL/NaAlg shells in centrifuge tubes (Fisher, UK) containing 40 mL of deionized water for 4 weeks by running three replicates. The meshes in the aqueous solution in the centrifuge tube were removed gently by tweezers. We provided a controlled environment (a fixed temperature of 21 °C with 79% humidity) for drying of meshes on the benchtop and avoided using excessive heat or rapid drying methods, as they may induce structural damage to the meshes. We regularly monitored the drying process to ensure that it was progressing as intended, and we checked for any signs of deformation, distortion, or quality issues during the drying phase. After 6 h, we weighed the meshes. Subsequently, the remaining meshes were placed into the aqueous solutions after the experiment was concluded. Meshes were collected and dried at weekly intervals, and the changes in their weight were tracked as a function of immersion time (Figure S3). The degradation rate of each mesh was evaluated using eq 1
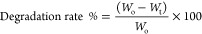
1where *W*_o_ and *W*_t_ are the weights (g) of the meshes before and after degradation, respectively.

Simultaneously, an electrical conductivity meter (WTW inoLab 720) was used each week to measure the conductivity of released ions in the aqueous solution. The mesh in the aqueous solution in the centrifuge tube was carefully taken by using tweezers and placed on a sterilized Petri dish. The remaining aqueous solution was filtered with sterilized filter paper and transferred to a beaker, which was previously washed with isopropanol. To ensure full mixing of the precipitated ions, the aqueous solution in the beaker was mixed with a sterilized magnetic stirrer at 300 rpm for 5 min. Subsequently, we utilized an electrical conductivity meter and after the experiments were concluded, meshes were transferred to the aqueous solutions. The overall electrical conductivity (EC; 491 μS/cm) was attributed to the release of Na^+^ ions upon degradation of the PCL/NaAlg shell (Figure S3b). This conductivity level was accompanied by a weight change in the PCL/NaAlg structure over a 4 week period, with a total decrease of 0.14 g (0.26 g at week 1 and 0.12 g at week 4,Figure S3a). To assess the permissible exposure of Na^+^ ions in the soil, the United States Salinity Laboratory (USSL) has established a classification system based on the electrical conductivity of the saturation extract (ECe) values. The measured value of 491 μS/cm is well below the threshold for level-1 (slightly saline-2000 μS/cm) classification.^36^ Therefore, we concluded that there will be no adverse impact on the soil at the observed conductivity level. The thermal degradation behavior of the PCL/NaAlg shells with 2.5, 5.73, and 9.5 NaAlg wt % was also tracked by thermal gravimetric analysis (TGA, NETZSCH STA 449C, Germany) to examine the effect of different NaAlg wt % on the degradation behavior. Samples were cut from the 3D-printed PCL/NaAlg shell (40 mg) and heated to 1000 °C at 25 °C/min under a 30 mL/min nitrogen flow (Figure S4).

### Release Profile of the C-Dots from the PCL/NaAlg Core–Shell System

The release profile of the C-dots was estimated through spectrofluorometry (Edinburgh Instruments FS5, UK) using Fluoracle software. The PCL/NaAlg shell with two different core inks, HPC/C-dots and HPC/PEG/C-dots, were immersed separately in centrifuge tubes containing 40 mL of deionized water for 4 weeks under ambient conditions by running three replicates of each. Per week, these meshes were gently collected using tweezers, and the aqueous solution in centrifuge tubes was mixed with a magnetic stirrer (∼300 rpm) for ∼5 min. Subsequently, all solutions were filtered with 0.45 μm pore size syringe filters (Sigma-Aldrich) and transferred to the fluorimeter cuvettes (Sigma-Aldrich) to monitor the weekly change in the emission intensity of the released C-dots as a function of the emission wavelength. Emission spectra were obtained by monitoring the wavelength range of 370–700 nm (λ_exc_ is 350 nm). After each measurement, the meshes and aqueous solutions in the fluorimeter cuvettes were transferred to the centrifuge tubes.

### Mechanical Characterizations of the Meshes

Three-point flexural tests were conducted according to the ASTM D790 standard using a Zwick mechanical tester (model Z100, Zwick/Roell, Germany). The bending specimens with different layers (2, 4, and 6) were printed in dimensions of 50.8 mm × 12.7 mm, having thicknesses less than 1.6 mm, with a span of 24.6 mm. The test used a 1 mm/min crosshead motion rate with 10 kN applied force. Additionally, we monitored the load-bearing capacity of the meshes. The meshes were fixed on the bending mandrel fixtures with tape, standard weights in the range of 10–500 g were gently placed onto the meshes, and the ability to withstand loads of different layers of meshes was observed. Tensile tests were performed according to the ASTM D638 standard using a Zwick mechanical tester (model Z100, Zwick/Roell, Germany). 10, 14.23, and 20 wt % PCL with 5.73 wt % NaAlg were cast into an ASTM D638 standard dog-bone-shaped mold and dried at room temperature for 24 h. The *Z*-axis moved at a constant speed of 5 mm/min with a 200 N force. All measurements were performed at room temperature.

### Preparation of Transparent Soil

The transparent soil was synthesized via the procedure described by Ma.^37^ A 1:4 mixture of NaAlg and phytagel was dripped into a MgCl_2_ solution with a polymer concentration of 1.2 wt %, and hydrogel-based transparent spherical beads were generated. A Murashige and Skoog basal medium solution was prepared using deionized water, and spherical beads were placed in this nutrient solution for 24 h. Then, the solution was filtered out, and the beads were collected (Figure S5).

## Results and Discussion

### Characterization of C-Dots

**Figure 1:**
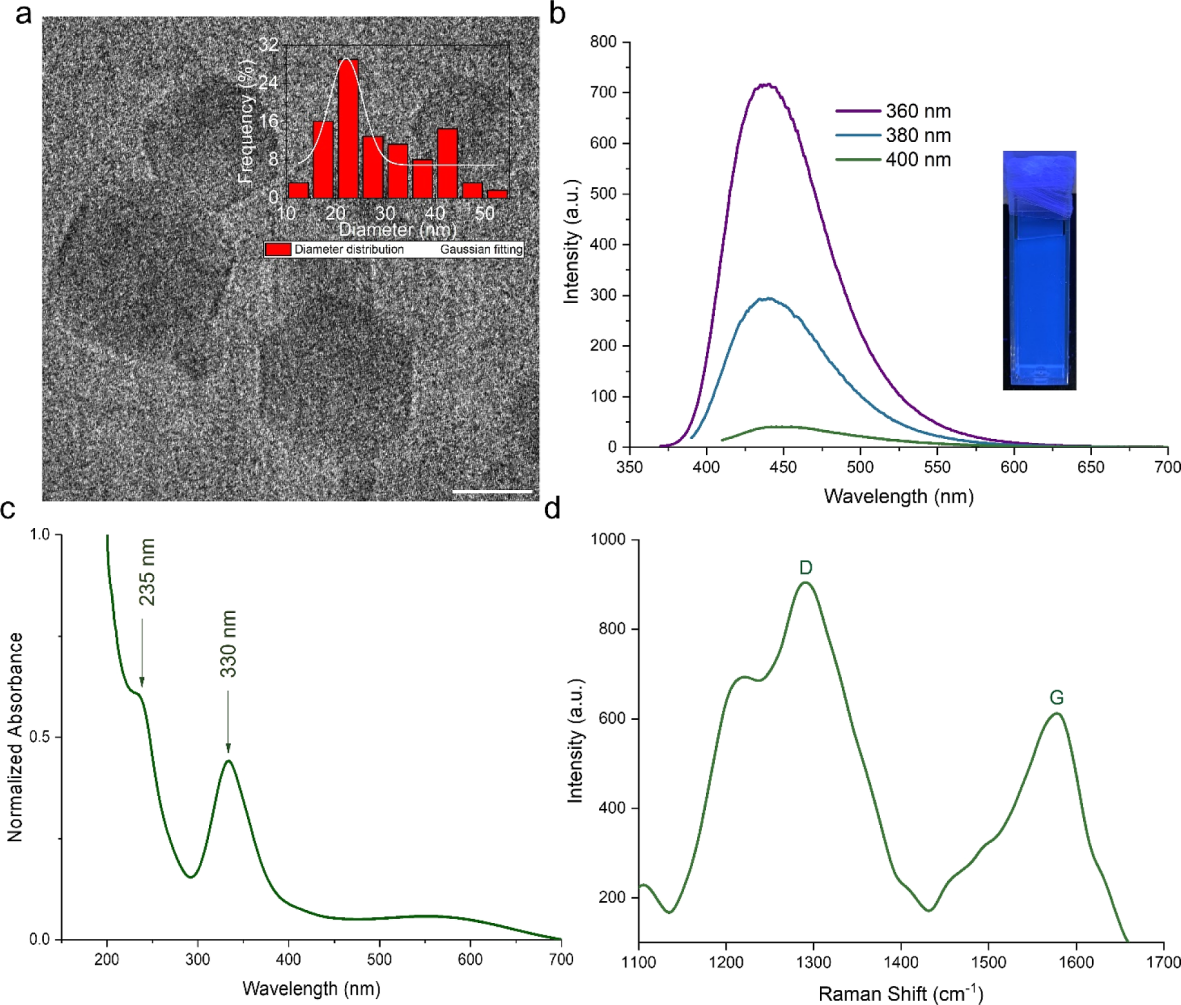
(a) TEM image of the unstacked spherical C-dots with particle size distribution in the range of 6–14 nm presented; the scale bar is 20 nm. The image is presented with the histogram of the size distribution of C-dots obtained from the TEM image, that is provided in the Supporting Information (Figure S1), (b) emission spectrum of C-dots at three different excitation wavelengths (360, 380, and 400 nm), the emission peak of the C-dots shifts from around 420 to 460 nm when the excitation wavelength changed, and (c) absorbance spectrum of C-dots, the first peak at 235 nm is assigned to π–π* transition (n is the non-bonding orbital and π* is the antibonding orbital) of C=C (sp^2^/crystalline core part) and the second peak at 330 nm is n−π* transition (π is the bonding orbital) of C–N/C=N/C=O, and (d) C-dot peaks at wavenumbers of 1300 and 1580 cm^–1^ correspond to the D (defect) and G (graphitic) bands of carbon, respectively.

In Figure 1c, the UV–vis spectrum exhibits a shoulder peak at 235 nm and a broad peak with its center at 330 nm, which is consistent with a typical C-dot absorption.^35^ The absorption spectrum further demonstrates that the C-dots possess higher absorption capacity in the UV range (200–400 nm) when compared to chloroplasts. In contrast, C-dots demonstrate minimal absorption between 400 and 700 nm. This observation substantiates that the incorporation of C-dots enhances the absorption of UV light by chloroplasts, without impeding the absorption of visible light. Consequently, this facilitation of UV light absorption promotes the enhancement of light conversion efficiency. As-prepared C-dots exhibited an excitation-dependent emission behavior, with a red shift observed in emission wavelengths as the excitation wavelength was increased. The Raman spectrum showed two distinct Raman bands at 1300 and 1580 cm^–1^, which can be attributed to sp^3^ defects (D-band) and sp^2^ carbon (G-band) in the synthesized N-doped C-dots. The D-band represents the vibrations of carbon atoms with dangling bonds on the disordered graphite termination plane. On the other hand, the G-band corresponds to the E_2g_ mode of graphite, indicating vibrations of sp^2^ carbon atoms in a two-dimensional hexagonal lattice. The peak intensities of the D-band and G-band were marked as *I*_D_ and *I*_G_, respectively. The measured intensity ratio (*I*_D_/*I*_G_) for the synthesized N-doped C-dots was approximately 1.2, suggesting the presence of a greater number of defective surface sites, which can be attributed to N doping. Additionally, the fluorescence observed in the synthesized C-dots can be assigned to the surface defects that trap the excited-state energy. These defects play a significant role in the emission properties of the C-dots, contributing to their distinctive fluorescence characteristics.

### Optimizations of the Flow Behavior of Core and Shell Inks for 3D Printing

In this work, we aimed to produce a mesh system that is degradable, flexible, and mechanically robust to deliver C-dots. To ensure the proper printing and encapsulation of C-dots in a degradable shell, core and shell inks should be able to produce continuous fibers without clogging the nozzle. Degradable polymers such as poly(lactic acid) (PLA),^38^ poly(glycolic acid) (PGA),^39^ and PCL^40^ are preferred for long-term degradation due to their higher hydrophobicity. Among these, PCL exhibits better mechanical properties,^41^ and degradation products of PCL are generally non-hazardous and they do not pose significant risks to soil organisms or ecological systems.^42,43^ However, the degradation of PCL in the pristine form was shown to take about 2–3 years, depending on the degradation mechanism.^41,44^ The degradation rate of PCL can be tuned by utilizing natural additives that increase the water uptake of PCL such as gelatin,^45^ chitosan,^46^ and NaAlg.^47^ We have chosen NaAlg to adjust the degradability of PCL due to its straightforward printability at room temperature and solubility in water without additional processing steps.

**Figure 2:**
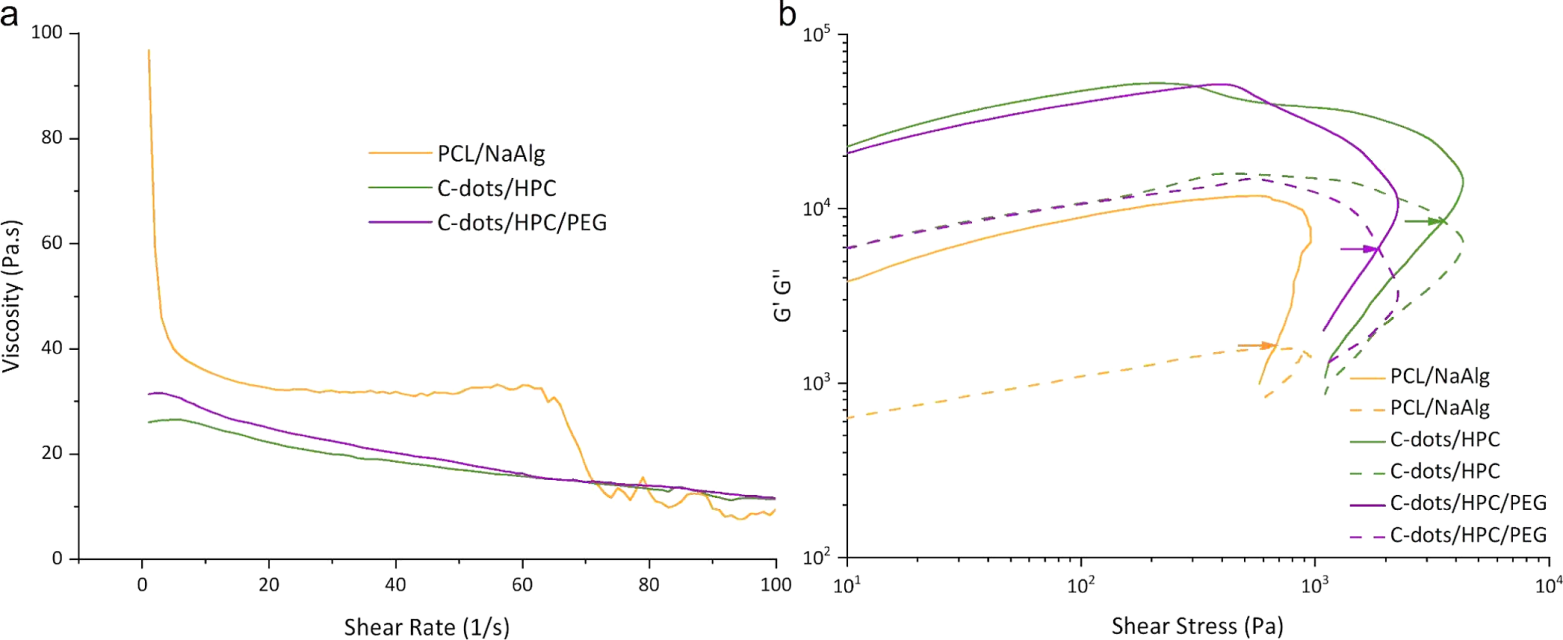
(a) Viscosity as a function of the shear rate of core and shell inks and (b) oscillatory rheological measurements (frequency = 10 rad s^–1^) of the shell and core inks. The yield stress of the inks was determined as the crossover point of storage and loss modulus, *G*′ = *G*″, and marked with an arrow.

We examined the rheology and printability of the inks that are composed of PCL and NaAlg in the range of 13–20 and 1.5–8 wt %, respectively. We determined the lower and upper limits of the PCL content based on the printing performance of the PCL and NaAlg mixture. Formulations that contain over 20 wt % PCL led to viscosities that required pressures that were above the printing capabilities of our 3D printer, while those with a PCL content below 13 wt % did not generate self-supporting and homogeneous fibers. We set the lower limit of NaAlg based on the printability and the minimum required amount that can accelerate the degradation rate of PCL. In contrast, the upper limit was based on reducing its detrimental effects on the mechanical properties of the mesh and the extrudability of the shell ink. A PCL-to-NaAlg ratio of ∼2.5:1 (14.32 wt % PCL and 5.73 wt % NaAlg) produced soft, continuous, robust, and highly uniform fibers without clogging the nozzle during printing (Figure 2). The printed mesh kept its mechanical integrity, yet significant degradation was observed within 2 weeks, which covers the seedling period for *T. aestivum* L. in transparent soil. The details of the non-optimal PCL/NaAlg mixtures are provided in Table S1.

We formulated the core ink to host the C-dots by fixing the concentration of C-dots at 0.1 mg/mL, which was reported to be in the beneficial range for plant growth.^13−15,21^ A viscosity modifier, PEG, was added at increasing amounts to this aqueous solution to tune the viscosity and ensure compatibility with the shell. PEG is known to improve the solubility of C-dots in aqueous solutions^48,49^ and in alignment with the previous reports,^45^ PEG200 exhibited a better printing performance compared to higher molecular weights (600 and 1000), which hindered the flow and clogged the nozzle. After evaluating the printing performance of various weight percentages of PEG200 (3–20 wt %) in the C-dot/PEG200 solution, we selected a C-dot/PEG200 ratio of 1:7.25 (0.69–5 wt %). However, ∼1 h after printing, the printed mesh structure of C-dot/PEG200 failed to preserve its shape due to the swelling of the mesh. Consequently, an alternative viscosity modifier, HPC, was introduced to the core ink in the range of 5–35 wt % to adjust the viscosity. HPC alone also achieved a consistent flow without swelling at 30 wt %, and it exhibited better mechanical integrity than the one with PEG. Therefore, we opted out to demonstrate two core ink systems, (i) C-dots/HPC/PEG200 and (ii) C-dots/HPC, in our analysis (Table 1).

**Table 1:** Optimized Formulations of the Shell and Two Core (C-1 and C-2) Inks (wt %)

shell	PCL/NaAlg	core	C-1	C-2
PCL	14.32	C-dots	0.69	0.69
NaAlg	5.73	HPC	30.00	25.00
chloroform	28.60	PEG200	-	5.00
water	37.24	water	69.31	69.31

The rheological response of the optimized inks was evaluated by measuring their apparent viscosity, yield stress, storage, and loss moduli. Expectedly, the viscosity of the inks decreased with an increasing shear rate and exhibited shear thinning behavior (Figure 2a). The flowability of inks improves when the shear stress exceeds the yield stress, while reasonable yield stress is desirable for shape retention after printing. For the ink to flow, the shear stress caused by the nozzle must be above yield stress. The oscillation stress sweep mode was used to determine the yield stress of the inks (Figure 2b). The storage (*G*′) and loss modulus (*G*″) of the inks were measured at 25 °C, and the intersection (*G*′ = *G*″, where the viscoelasticity changed from linear to non-linear) was defined as the yield stress. The yield stress point was used to determine the critical condition for shear flow during 3D printing. The yield stress of PCL/NaAlg, C-dots/HPC, and C-dots/HPC/PEG200 was 678.9, 3589.3, and 1865.8 Pa, respectively (shown by arrows in Figure 2b). The shear stress at the inner nozzle wall was estimated as 4196, 5292, and 3175 Pa using eq 2
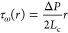
2where Δ*P* (Pa) is the applied pressure, *L*_C_ (m) is the length of the inner nozzle, and *r* (m) is the core nozzle radius.

The yield stress of all inks was below the maximum shear stress at the nozzle wall. The rheological behaviors of inks can substantially affect their printing performance. Shear thinning limits the entanglement of polymer chains and enables the ink to flow easily with less resistance at higher shear rates. Therefore, we estimated the printing parameters (e.g., speed and pressure) based on the rheological characterization of the optimized inks to eliminate possible defects (e.g., clogging, discontinuous printing, and sagging) during printing. The pneumatic pressure and nozzle speed were coordinated synchronously to deliver continuous filaments and fixed strut diameter. With increasing pressure (from ∼21 × 10^–9^ to 50 × 10^–9^ Pa for the core and from ∼14 × 10^–9^ to ∼36 × 10^–9^ Pa for the shell until the sudden release of the ink) in the process, the strut diameters were enlarged. In contrast, with the increasing speed of the nozzle (from 1 to 5 mm/s), the inks came out as droplets, and the strut diameter decreased. Therefore, to obtain a homogeneous strut diameter, we maintained a constant nozzle speed of 2 mm/s, while the applied pressure was fixed to ∼36 × 10^–9^, ∼21 × 10^–9^, and ∼14 × 10^–9^ Pa for C-dots/HPC, C-dots/HPC/PEG200, and PCL/NaAlg, respectively.

### Morphological Characterization of the Meshes

**Figure 3:**
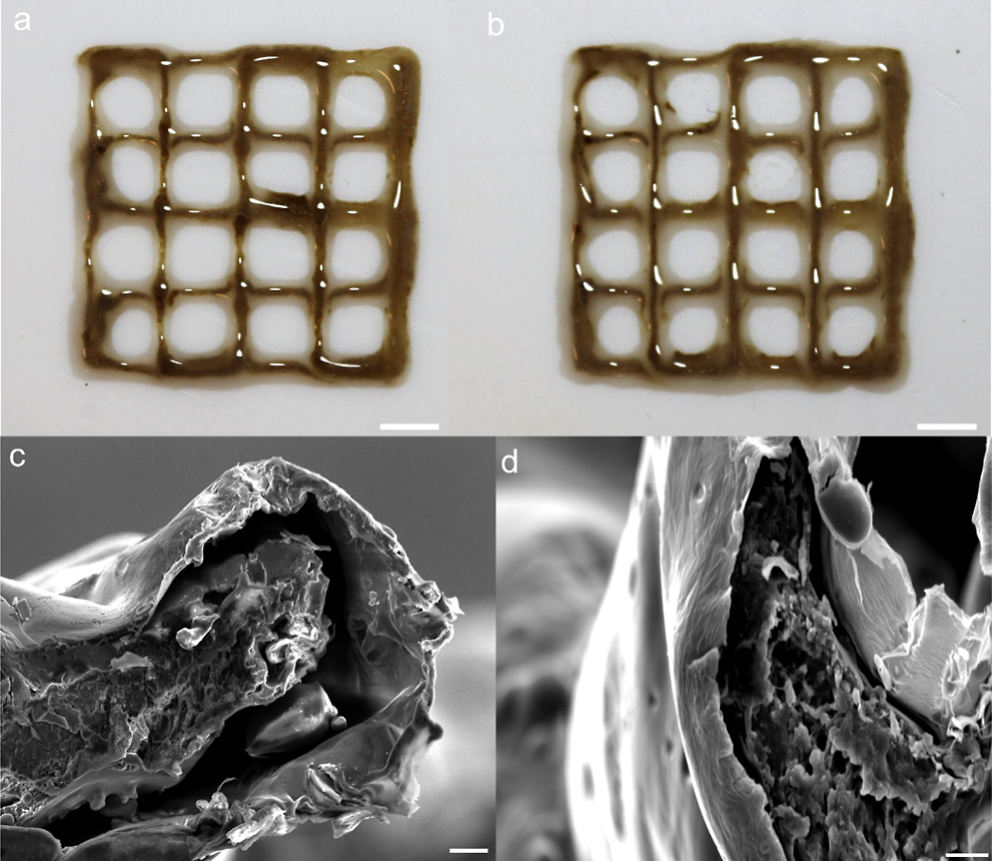
Pictures of 3D-printed meshes, PCL/NaAlg shell with (a) C-dot/HPC and (b) C-dot/HPC/PEG200 core inks, taken directly after printing; the scale bar is 5 mm; SEM images of the cross sections of the printed meshes, C-dots in (c) HPC and (d) HPC/PEG200; the scale bar is 100 μm.

Figure 3c,d shows the cross-sectional morphology of 3D-printed meshes of encapsulated C-dot inks with the PCL/NaAlg shell under SEM. The textural contrast between the core and shell layers was clear. The core diameters for C-dots with HPC and HPC/PEG200 were ∼0.55 and ∼0.70 mm, respectively, whereas the shell thickness was ∼0.22 mm in both cases. These dimensions match the inner and outer diameters of the nozzle we used for printing (15/19-gauge nozzle: 15G—inner diameter (ID): 1.40 mm, outer diameter (OD):1.80 mm, 19G—ID:0.67 mm, OD:1.07).

### Mechanical Properties of the Meshes

**Figure 4:**
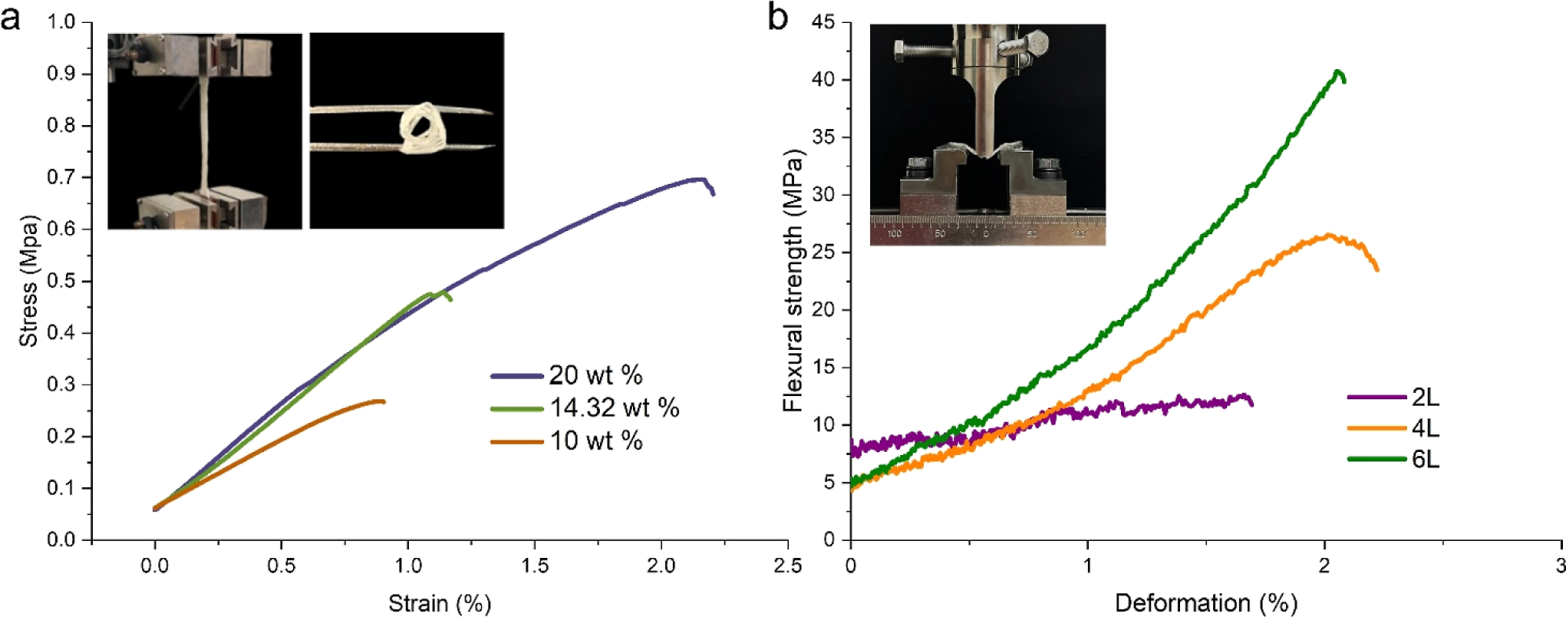
(a) Tensile test for 5.73 wt % NaAlg with 10, 14.32, and 20 wt % PCL; the inset of the images shows a sample during a tensile test (left) and a foldable PCL/NaAlg mesh (right), and (b) three-point bending test for 5.73 wt % NaAlg with 14.32 wt % PCL with a different number of layers; the inset of the image displays a three-point flexural test.

The typical tensile stress–strain curves of PCL-NaAlg samples containing 10, 14.32, and 20 wt % PCL are displayed in Figure 4a. Expectedly, the specimen with 20 wt % PCL exhibited the largest level of strength (∼0.7 MPa), while the specimen with 10 wt % PCL showed minimum tensile strength (∼0.3 MPa). The typical stress–strain curves of different layers of PCL-NaAlg meshes under flexural loading are presented in Figure 4b. We characterized two, four, and six layers of printing of the shell ink with 14.32 wt % PCL and 5.73 wt % NaAlg. The six-layer PCL/NaAlg meshes had the largest flexural strength, ∼40 MPa, while the lower number of layers produced lower flexural stress values.

### Release Behavior of C-Dots and Kinetic Studies

To assess the influence of the environment on the degradation of polymer shells, two different media were employed to investigate the release pattern of C-dots, deionized water, and lab-made transparent soil. In the presence of water, NaAlg undergoes swelling, creating additional space and pathways within the PCL matrix where water molecules can further permeate the structure.^50^ Water molecules hydrolyze the ester linkages of PCL, leading to the degradation of the mesh. While the mesh structures were degraded to half of their weight after being immersed in deionized water over a month, they underwent complete degradation within 15 days when placed in the transparent soil. Transparent soil served as a porous medium with ideal optical properties and stability to support plant growth comparable to natural soil.^37^ The difference in the degradation behavior of the PCL shell can be attributed to external factors such as microbial activity and the chemical composition of the transparent soil. Furthermore, the presence of metal ions within the boric acid in the Murashige and Skoog solution serves as catalysts and effectively enhances the degradation process.^51,52^ To assess the proximity of the transparent soil to the common agricultural applications, we conducted an experiment using natural soil from the ion-deficient region of Eskisehir, Türkiye (pH of 8.0 and low organic matter content of ∼1 %). The PCL/NaAlg meshes demonstrated ∼45 wt % degradation after a 4-week period in natural soil (Figure S13).

**Figure 5:**
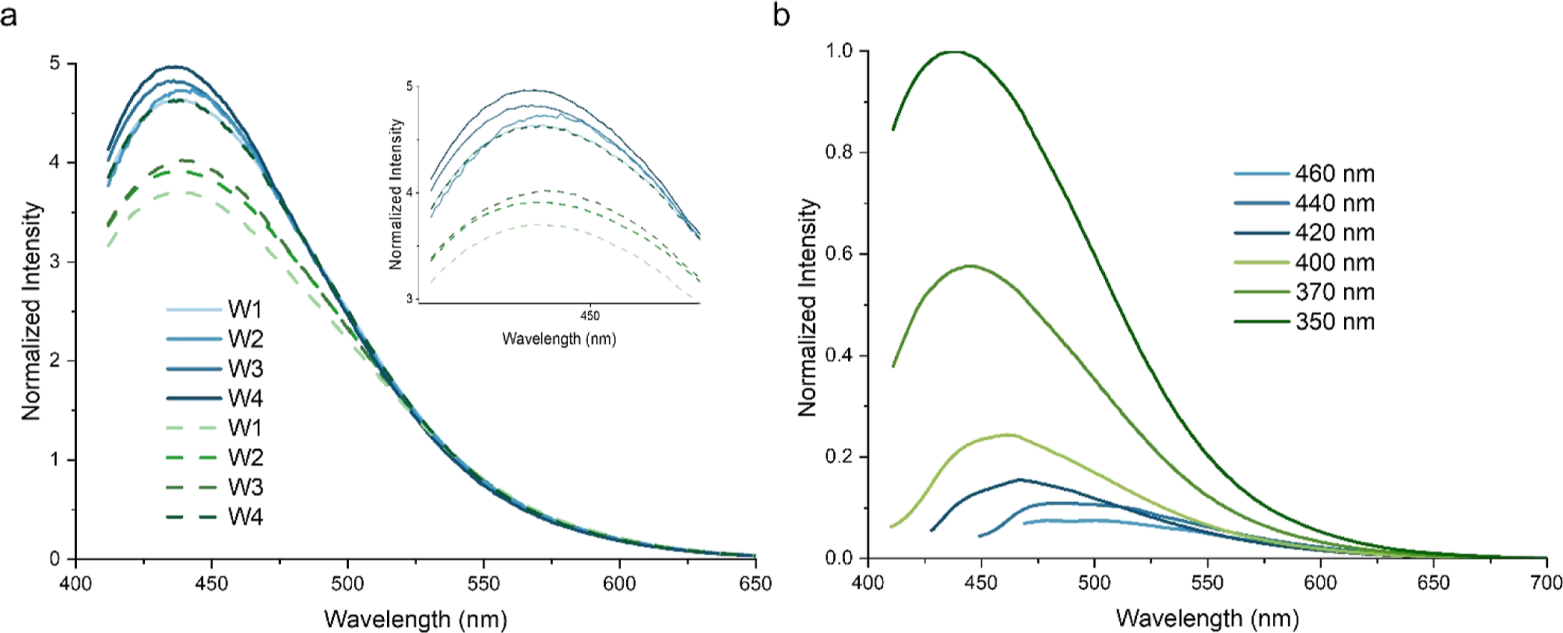
(a) Normalized fluorescence intensity of the C-dots divided by the median intensity. The solid line corresponds to the released C-dots into the aqueous solution from meshes of C-dots/HPC, whereas the dashed line represents the released C-dots into the aqueous solution from meshes of C-dots/HPC/PEG200 with PCL/NaAlg for 4 weeks. Furthermore, an inset image is provided to offer a magnified view of the main plot, enabling a more detailed analysis of the weekly change of intensity and (b) excitation-dependent emission behavior of the released C-dots from the C-dot/HPC-PCL/NaAlg matrix to the aqueous solution at W4.

C-dots that were released into the aqueous solution and transparent soil were tracked by spectrofluorometry and confocal microscopy, respectively. The emission wavelength and intensity are sensitive to the concentration of C-dots in an aqueous solution, which depends on the degradation and release period.^53^ The spectrofluorometry recorded the emission intensity of the released C-dots as a function of emission wavelength to analyze the release profile of C-dots for 4 weeks. Normalized emission intensity spectra showed a broad emission peak with a λ_max_ around 440 nm (λ_exc_ with 350 nm) for all samples from week 1 (W1) to 4 (W4) (Figure 5). The increase in emission intensity indicated the successful degradation of 3D-printed meshes, leading to the subsequent release of C-dots.

The emission spectra of C-dots, released from the shell matrix, are depicted in Figure 5b, demonstrating a significant overlap with the absorption spectra of chlorophyll *a* and *b* (Figure S14) in the blue light region (400–500 nm). Combined with the absorption spectrum of C-dots in Figure 1c, it indicates that C-dots possess the potential to convert UV light into visible light, leading to an improved light utilization capability for chloroplasts by providing a suitable range of visible light that chloroplasts can efficiently absorb. Furthermore, the introduction of N-doped C-dots can facilitate chlorophyll formation due to the critical role of nitrogen as a component of chlorophyll. Augmenting the chlorophyll content in plants is beneficial for enhancing light absorption efficiency and increasing carbohydrate production during photosynthesis.^13−15,50^ Moreover, previous studies^51^ have reported the effective ability of C-dots to enhance the separation efficiency of photogenerated electron–hole pairs and facilitate the rate of electron transfer, ultimately leading to improved photosynthesis.

### Translocation of C-Dots and Assessment of Plant Growth

**Figure 6:**
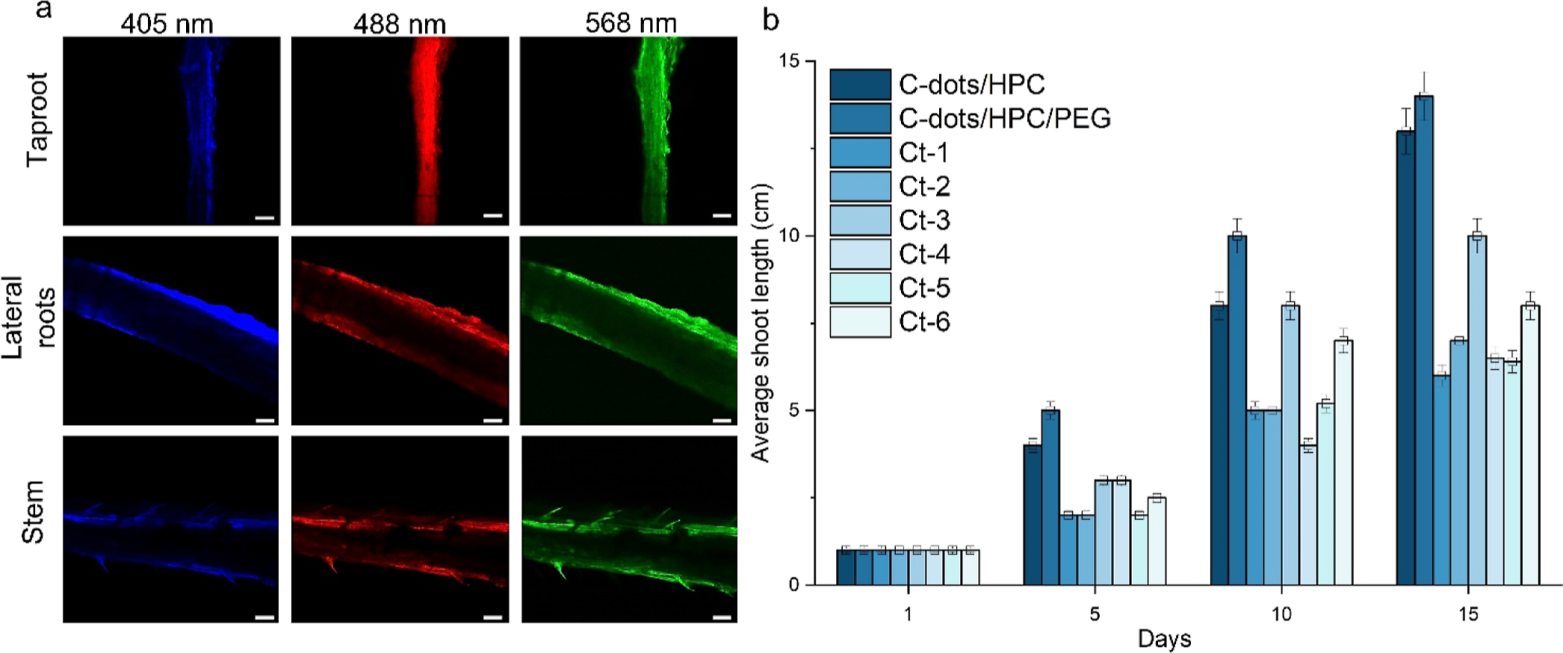
(a) Day 16 confocal microscopy images of tissues of *Triticum aestivum* L. seed growth with C-dot/HPC-PCL/NaAlg mesh, the scale bar is 50 μm, and (b) shoot length of the plants was measured in three replicates throughout the experiment. Initially, on day 1, all the plants were of a similar length. Subsequently, measurements were taken at 5 day intervals until day 15, resulting in a total of four measurements.

Due to their water solubility and size, C-dots can transport easily in an aqueous environment. Combined with their dispersibility, through the capillary effect, they are able to diffuse into the growing plant body. Plants were exposed to 0.1 mg/mL C-dots, and the translocation of C-dots was tracked using confocal microscopy with three replicates. Essentially, over a 15-day period, the degradation of PCL/NaAlg shell meshes, which contained the C-dots/HPC and C-dots/HPC/PEG, resulted in the release of C-dots into the soil. On day 16, plants were dissected, separating the tissues to the taproot, lateral roots, and stem, which were placed individually on confocal Petri dishes for examination. The uptake of C-dots by *T. aestivum* L. was identified in vivo, through blue (410–507 nm), green (494–601 nm), and red (568–712 nm) luminescent emissions from C-dots observed at 405, 488, and 568 nm excitation, respectively. The fluorescence signals from C-dots exhibited a strong correlation with the images of *T. aestivum* L. tissues, demonstrating the effectiveness of fluorescent labeling and the translocation of C-dots. The blue, green, and red fluorescent channels consistently exhibited stronger fluorescent intensity at the edges and relatively weaker intensity in the central regions of the taproot, lateral roots, and stem across both samples. This observation was attributed to the accumulation of C-dots in these areas. The notable distinction observed was that the plant cultivated with the C-dot/HPC/PEG200 mesh exhibited a higher signal intensity (Figure 6a) in comparison to the plant grown with the C-dot/HPC mesh (Figure S12). Such difference in the fluorescence signal can be due to the enhanced stability and better dispersion of C-dots facilitated by the presence of PEG200. PEG is a hydrophilic polymer with excellent solubility in water and other polar solvents and is recognized for its ability to improve the solubility and dispersibility of C-dots in aqueous solutions.^48,49^ When PEG is added to a C-dot dispersion, it would act as a stabilizing agent and prevents the aggregation or agglomeration of C-dots. PEG molecules form a protective layer around the C-dots, contributing to particle stability due to a steric hindrance. This steric stabilization effect inhibits the C-dots from coming into close contact and forming larger aggregates. As a result, the C-dots remained dispersed and well-distributed within the medium. Moreover, through confocal microscopy analysis of the neat plant (plant with no treatment), only a few green and red fluorescence signals were detected, exhibiting a patchy distribution pattern. Conversely, no fluorescence signal was identified in the blue fluorescence channel (Figure S12d), indicating a probable occurrence of autofluorescence originating from the *T. aestivum* L.^54−56^ Overall, the samples containing C-dots demonstrated strong fluorescence signals, indicating a well-distributed presence of C-dots within the taproot, lateral roots, and stem of *T. aestivum* L.

The degradation rate of polymers and the effect of released C-dots on plant growth were correlated with the shoot and root length of the plant (cm). Each treatment group comprised three replicates, wherein each individual replicate corresponded to an independent plant subjected to its respective treatment condition. Descriptive statistics, encompassing both means and standard deviations, were computed for each treatment group, focusing on the measured shoot length across a span of 15 days. The mean shoot length values for plant groups exposed to C-dots-HPC and C-dots-HPC-PEG200 were determined as 13.4 ± 0.52 and 11.43 ± 4.46 cm, respectively. The mean values and standard deviations of control plants are given in Table S4. Plants treated with C-dots/HPC and C-dots/HPC/PEG200 grew ∼12 and ∼13 cm in 15 days, respectively, while Ct-1 (plant with no treatment) had the lowest shoot length of ∼5 cm, which underlines the boosting effect of C-dots on plant growth. Additionally, seeds were separately treated with the meshes without C-dots to observe the effects of each polymer on plant growth. Out of all the mesh treatments, Ct-3 (HPC/PEG200) resulted in the longest shoot lengths, reaching ∼9 cm, which is still 1.4-fold shorter than the plant that was treated with C-dots. On the other hand, when the core materials were separated, Ct-6 (PEG200) exhibited a growth of ∼7 cm, while Ct-5 (HPC) displayed ∼5 cm. This suggests that the co-existence of PEG200 and HPC in the core have promoted plant growth. Treatment with a mesh containing a shell formulation Ct-4 (PCL/NaAlg) showed a shoot length of ∼5.5 cm, and the shoot length of Ct-2 (PCL/NaAlg-HPC/PEG200) was slightly longer, ∼6 cm. Overall, we concluded that the presence of C-dots elevated plant growth between 1.4- and 2.5-fold compared to the control groups, and in alignment with previous reports,^58−60^ the presence of PEG, HPC, and NaAlg also contributed to the growth.

## Conclusions

In this work, we demonstrated a room-temperature, mechanical route for the fabrication of a controlled release system for as-prepared C-dots through coaxial printing. We designed inks to encapsulate C-dots in the core and offer a biodegradable shell to exploit the photosynthetic efficiency of C-dots for plant growth. The analysis of the degradation profile of 3D-printed meshes showed complete degradation within approximately 4 weeks when exposed to both water and natural soil environments, but when the meshes were in transparent soil, the degradation process occured within a shorter timeframe, typically around 15 days. We explored the growth of *T. aestivum* L. seeds in the presence of N-doped C-dots. These C-dots enhance light absorption efficiency in chloroplasts, displaying higher UV absorption compared to chloroplasts and minimal absorption in the visible range. As a result, chloroplasts experience increased UV light absorption without hindering visible light absorption, ultimately leading to improved light conversion efficiency. We monitored the uptake and distribution of C-dots in *T. aestivum* L. with confocal microscopy. Additionally, we measured the shoot length, revealing that C-dots facilitated a 2.5-fold increase in growth compared to plants with no treatment. These findings demonstrated that C-dots can serve as an effective nanofertilizer in agriculture. Moreover, C-dots enabled a non-toxic scheme to track chemical intake in a plant body, thus confirming that C-dots can be a good candidate for non-toxic in vivo biolabel owing to their fluorescent properties. Overall, using 3D printing technology, we successfully delivered C-dots and created sub-mm fibers in a mesh form. This approach resulted in a robust yet flexible frame with 100% encapsulation efficiency and minimal waste production (e.g., evaporation of chloroform). This eco-friendly encapsulation method has the potential to host other chemicals and generate agricultural textiles.

## Supporting Information Available

Optical microscopy images, degradation kinetics, images of seed germination and transparent soil, ink formulations, images of PCL/NaAlg ink at different PCL wt % ratios, images of unsuccessful 3D print examples, confocal microscopy images, TEM images, and absorption spectra of chlorophyll (PDF)
