# Supplementary material for: Encapsulation of Carbon Dots in a Core–Shell Mesh through Coaxial Direct Ink Writing for Improved Crop Growth
Source: ACS Sustain Chem Eng. 2023 Sep 11;11(38):13939–49. doi: 10.1021/acssuschemeng.3c02641 (PMC10523578; doi:10.1021/acssuschemeng.3c02641)
Supplement: Supplementary file 1 — sc3c02641_si_001.pdf [file sc3c02641_si_001.pdf]

Supporting Information

Encapsulation of Carbon Dots in a Core-Shell Mesh through Coaxial Direct Ink Writing for Improved Crop Growth

Isik Arel[†], Ayse Ay[†], Jingyi Wang[‡,†], Luz Karime Gil-Herrera [‡,†], Ahu Gümrah Dumanli [‡,†], and Ozge Akbulut[†]*

[†]Faculty of Engineering and Natural Sciences, Sabanci University, Tuzla, Istanbul 34956, Turkey

[‡]Department of Materials, The University of Manchester, Manchester, M13 9PL, United Kingdom, [†]Henry Royce Institute, The University of Manchester, Oxford Road, Manchester, M13 9PL, United Kingdom

Contents *Page number*

**Figure S1.** Bright-field TEM image and particle size analysis of the C-dots	*S3*
**Figure S2.** Dark-field optical images of the C-dots encapsulated with PCL/NaAlg	*S4*
**Figure S3.** Degradation kinetics study of the PCL/NaAlg, C-dots/HPC-PCL/NaAlg, and C-dots/HPC/PEG200-PCL/NaAlg constructs	*S5*
**Figure S4.** TGA curves of the PCL/NaAlg shell, with different wt % of NaAlg contents	*S6*
**Figure S5.** Digital photographs describing the seed germination and transparent soil	*S6*
**Figure S6.** Digital photographs from the plant-growth studies	*S7*
**Figure S7.** Digital photographs show plant growth studies in rows	*S8*
**Table S1.** Optimization studies to determine the range for the shell inks	*S8-10*
**Table S2.** Optimization studies to determine the range for the core inks without C-dots	*S10*
**Table S3.** Optimization studies to determine the range for the core inks with C-dots	*S11*
**Figure S8.** The qualitative flowability of PCL/NaAlg inks from digital photographs	*S12*
**Figure S9.** Digital photographs of the NaAlg/HPC meshes	*S12*
**Figure S10.** Digital photographs of the PCL/NaAlg meshes	*S13*
**Figure S11.** Digital photographs of the C-dots encapsulated in a) PEG/HPC-2, b) PEG-2, and c) PEG/HPC-1 with PCL/NaAlg shells constructs	*S14*
**Figure S12.** Fluorescence microscopy images of the plant treated with C-dots/HPC mesh, and column d is the control plant (no treatment).	*S15*
**Figure S13.** The degradation study of PCL/NaAlg from week one to four	*S16*
**Figure S14.** Absorption spectra of chlorophylls	*S16*
**Table S4.** Statistical analysis of plant growth studies	*S17*

TEM images of C-dots

The particle size and size distribution were determined by averaging more than 50 particles. Particle size distribution was in the range of 10–50 nm. The Gaussian fitting curves demonstrated that the average diameter of the corresponding products was 22.5 nm.

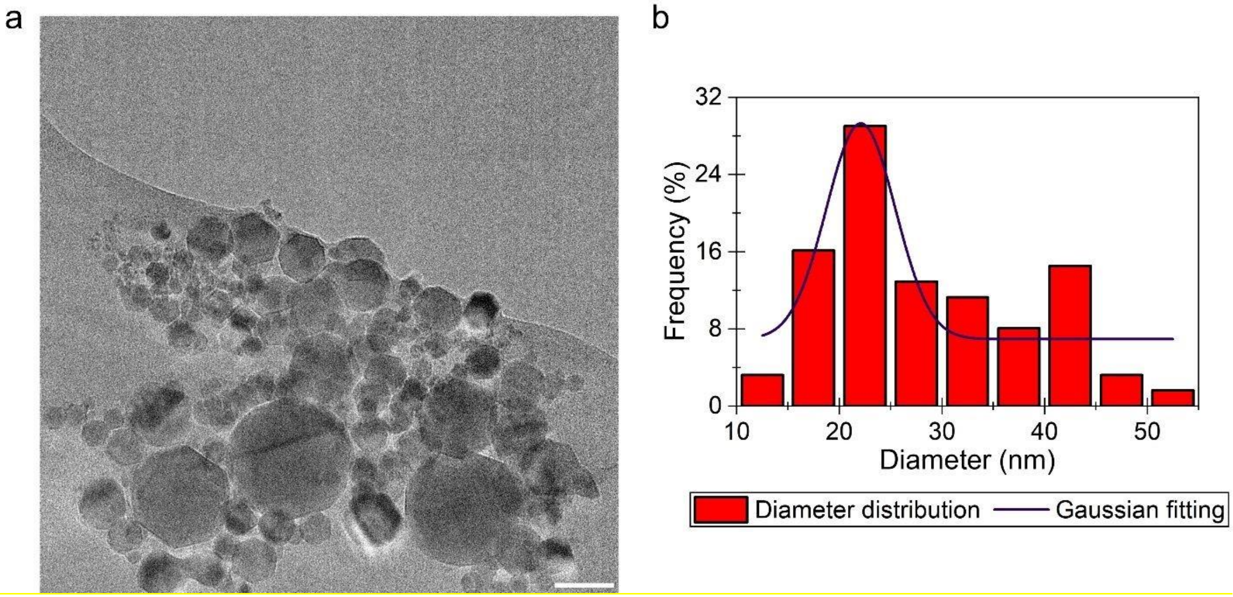

Figure S1. a) Transmission electron microscopy (TEM) images were obtained to illustrate the size distribution of the as-prepared C-dots, the scale bar is 50 nm and b) diameter distribution and Gaussian fitting curves

Optical microscopy

Optical microscopy was used to investigate the core-shell structure of the meshes. To image the cross-sections of the printed meshes, the samples were immersed in liquid nitrogen, and the filaments

were fractured by bending them. Images of various magnifications were taken at room temperature in the dark field mode. The microporous structure of PCL is due to the evaporation of chloroform.

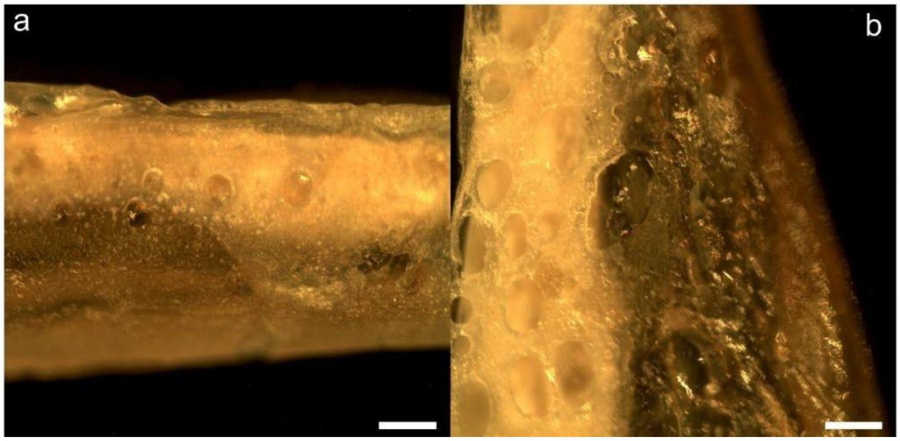

Figure S2. Dark-field optical image of the top view of C-dots encapsulated with PCL/NaAlg, a) 5x magnification and b) 20x magnification, scale bars represent 1 mm.

Degradation kinetics

Change in the weight of meshes was used to characterize the degradation behavior of the shell and the release behavior of the core. Additionally, the electrical conductivity (EC; μS/cm) of the aqueous solutions in falcons was measured as a function of immersion time.

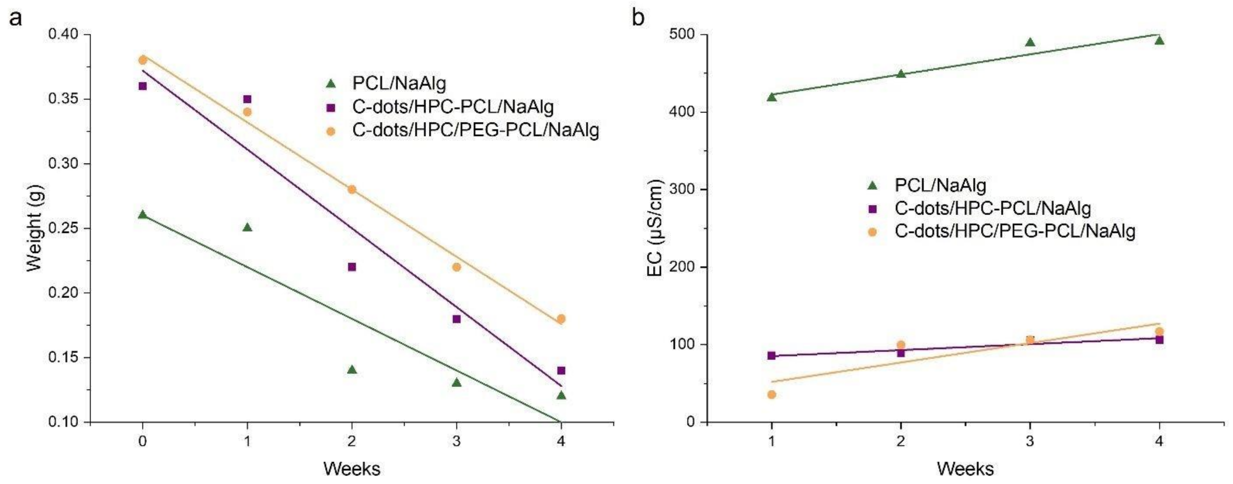

Figure S3. a) Weight change and b) normalized electrical conductivity measurements of PCL/NaAlg, C-dots/HPC-PCL/NaAlg, and C-dots/HPC/PEG200-PCL/NaAlg for 4 weeks in deionized water

The thermal degradation behavior of the PCL/NaAlg shell was studied by thermal gravimetric analysis (TGA) by keeping the PCL wt % at 14.32 while changing the NaAlg wt %. The thermal decomposition of PCL starts around ~400 °C. [1,2] The presence of NaAlg reduced the decomposition start point of PCL to ~200 °C, and about 90% of mass loss occurred until 400 °C. The different wt % of NaAlg did not lead to any significant changes in the decomposition start point.

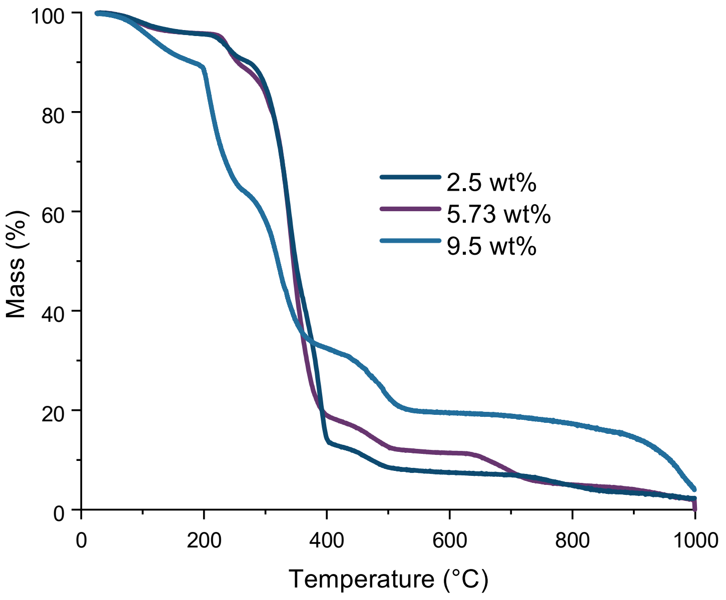

Figure S4. TGA curves of the PCL/NaAlg shell, with different wt % of NaAlg content

Images of seed germination, transparent soil, and plant growth

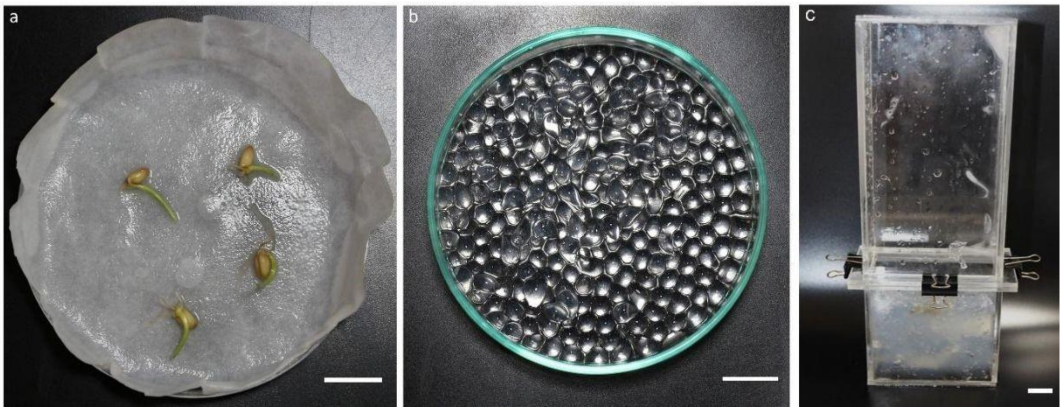

Figure S5. a) Seed germination of *Triticum aestivum* L., b) transparent soil as a growth medium, and c) a set-up for plant growth that contains germinated seeds, transparent soil, and 3d printed mesh in a controlled environment. Both transparent soil and set-up allowed the monitoring of seed growth. Scale bars represent 2.5 cm in images a, b, and 1 cm in c.

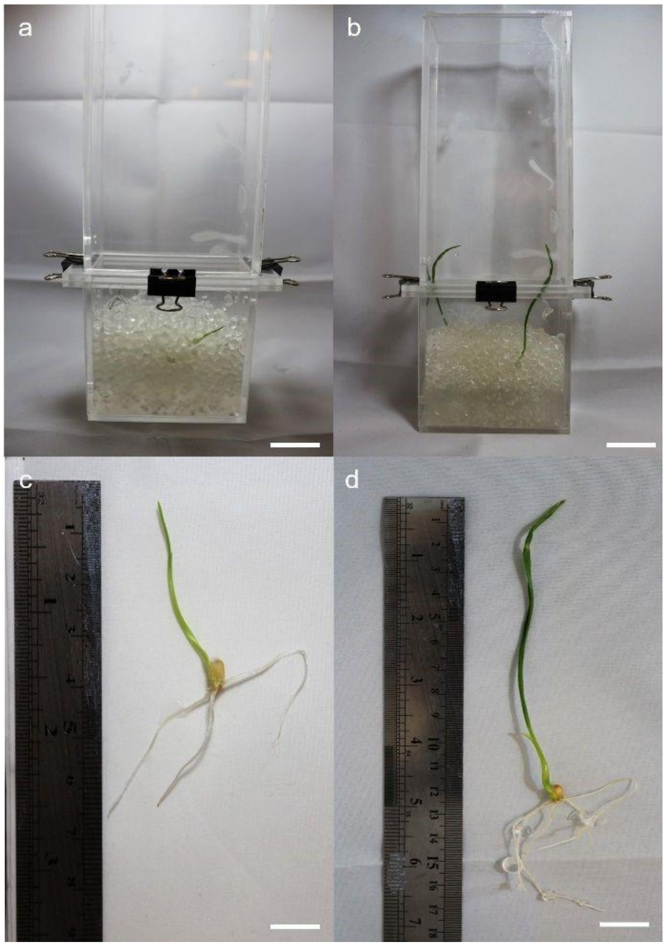

Figure S6. Plant-growth and set-up with C-dots/HPC mesh; a and c) day-1, b and d) day-15

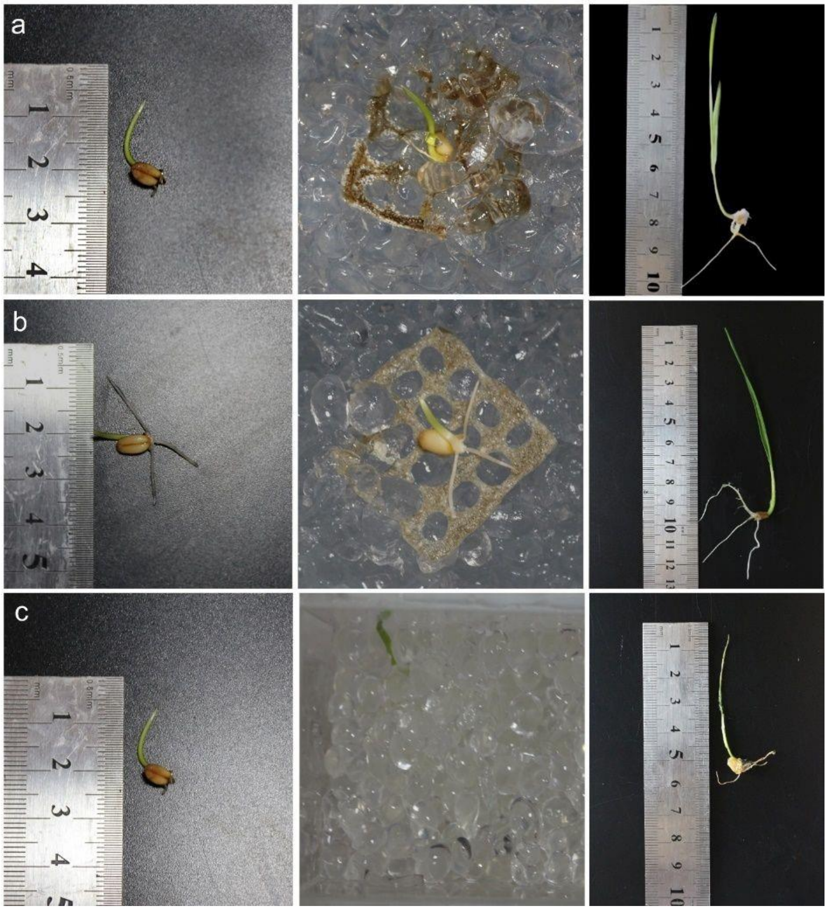

Figure S7. The images show plant growth in rows; a) C-dots/HPC, b) C-dots/HPC/PEG, and c) control, no mesh.

Ink formulations

Table S1. Optimization range for the shell inks

Mixture	Polymer 1	wt %	Polymer 2	wt %	Polymer 3	wt %
PCL-1	PCL	5	-	-	-	-

PCL-2	PCL	10	-	-	-	-
PCL-3	PCL	15	-	-	-	-
PCL-4	PCL	20	-	-	-	-
PCL-5	PCL	22	-	-	-	-
PCL-6	PCL	25	-	-	-	-
NaAlg/HPC-1	NaAlg	10.0	HPC	3.3	-	-
NaAlg/HPC-2	NaAlg	12.7	HPC	2.5	-	-
NaAlg/HPC-3	NaAlg	12.9	HPC	0.8	-	-
NaAlg/HPC-4	NaAlg	18.7	HPC	6.3	-	-
NaAlg/HPC-5	NaAlg	21.4	HPC	7.1	-	-
NaAlg/HPC-PCL-1	NaAlg	5.6	PCL	19.7	HPC	0.4
NaAlg/HPC-PCL-2	NaAlg	6.6	PCL	16.4	HPC	1.63
PCL/NaAlg-1	NaAlg	6.7	PCL	16.6	-	-
PCL/NaAlg-2	NaAlg	1.5	PCL	18.3	-	-
PCL/NaAlg-3	NaAlg	8.7	PCL	14.4	-	-
PCL/NaAlg-4	NaAlg	6.7	PCL	13.4	-	-
PCL/NaAlg-5	NaAlg	5.73	PCL	14.32	-	-
PCL/NaAlg-6	NaAlg	5.73	PCL	20	-	-

PCL/NaAlg-7	NaAlg	4	PCL	22	-	-
PCL/NaAlg-8	NaAlg	9.5	PCL	14.32	-	-
PCL/NaAlg-9	NaAlg	2.5	PCL	14.32	-	-
PCL/NaAlg-10	NaAlg	5.73	PCL	10	-	-
PCL/NaAlg-11	NaAlg	5.73	PCL	20	-	-

Table S2. Optimization range for the core inks without C-dots

Mixture	Polymer 1	wt %	Polymer 2	wt %
PEG/HPC-1	PEG200	16.3	HPC	6.55
PEG/HPC-2	PEG200	5	HPC	20
PEG/HPC-3	PEG200	5	HPC	3.5
PEG/HPC-4	PEG200	5	HPC	25
PEG/HPC-5	PEG200	3	HPC	2
PEG/HPC-6	PEG200	12	HPC	5.9
PEG/HPC-7	PEG200	5	HPC	30
PEG/HPC-8	PEG200	15	HPC	20
PEG/HPC-9	PEG200	20	HPC	10
HPC-1	-	-	HPC	5
HPC-2	-	-	HPC	10
HPC-3	-	-	HPC	15

HPC-4	-	-	HPC	20
HPC-5	-	-	HPC	25

Table S3. Optimization range for the core inks with C-dots

Mixture	Polymer 1	wt %	Polymer 2	wt %	wt % C-dots
PEG-1	PEG2000	3	-	-	0.69
PEG-2	PEG200	5	-	-	0.69
PEG-3	PEG200	15	-	-	0.69
PEG-4	PEG200	20	-	-	0.69
HPC-1	HPC	5	-	-	0.69
HPC-2	HPC	20	-	-	0.69
HPC-3	HPC	25	-	-	0.69
HPC-4	HPC	30	-	-	0.69
HPC-5	HPC	35	-	-	0.69
PEG/HPC-1	PEG200	5	HPC	15	0.69
PEG/HPC-2	PEG200	5	HPC	20	0.69
PEG/HPC-3	PEG200	5	HPC	25	0.69

Images of PCL/NaAlg ink with different PCL wt %

To demonstrate the importance of ink viscosity in 3d printing, we inverted the vials that contained various weight percentages of PCL. 10 wt % of PCL showed free-flowing behavior as the vial was turned upside down; in contrast, the vial that contained 20 wt % of PCL did not exhibit any flow even after being inverted for 10 seconds.

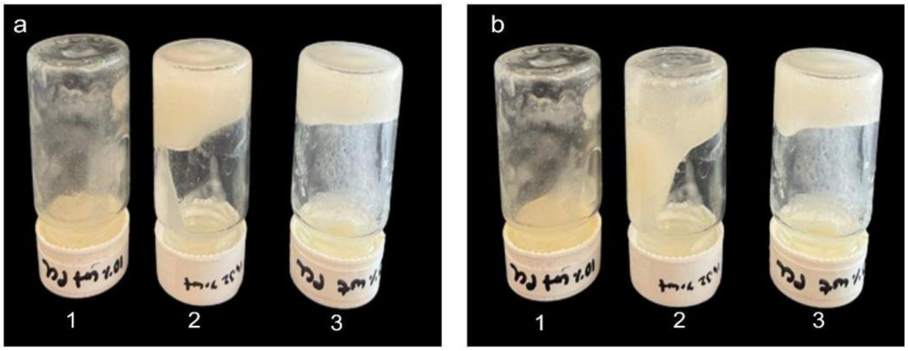

Figure S8. The qualitative flowability of PCL/NaAlg inks: 1: 10 wt % PCL, 2: 14.32 wt % PCL, 3: 20 wt % PCL, a) right after inversion of the vial and b) 10 seconds later

Unsuccessful 3d print examples with different wt % ratios and printing parameters

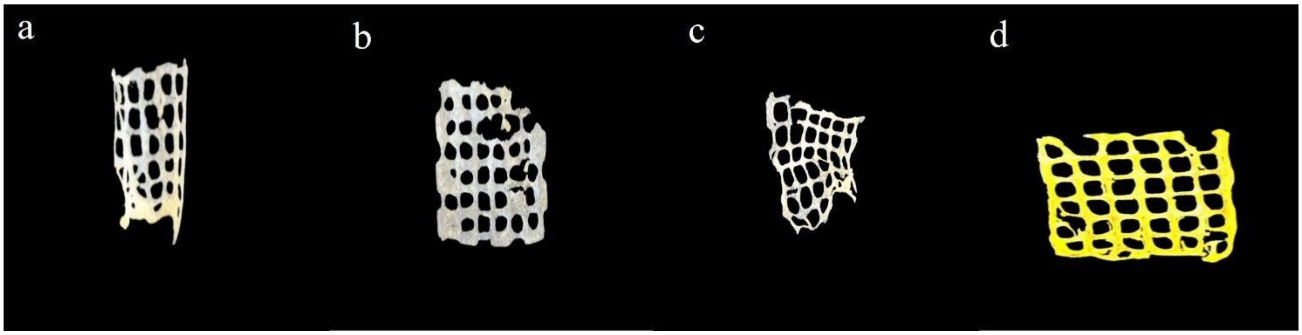

Figure S9. NaAlg/HPC meshes a) NaAlg/HPC-5, b) NaAlg/HPC-4, c) NaAlg/HPC-2, and d) NaAlg/HPC/PCL-1, the yellow color is due to the use of Pluronic F-127 as a core ink.

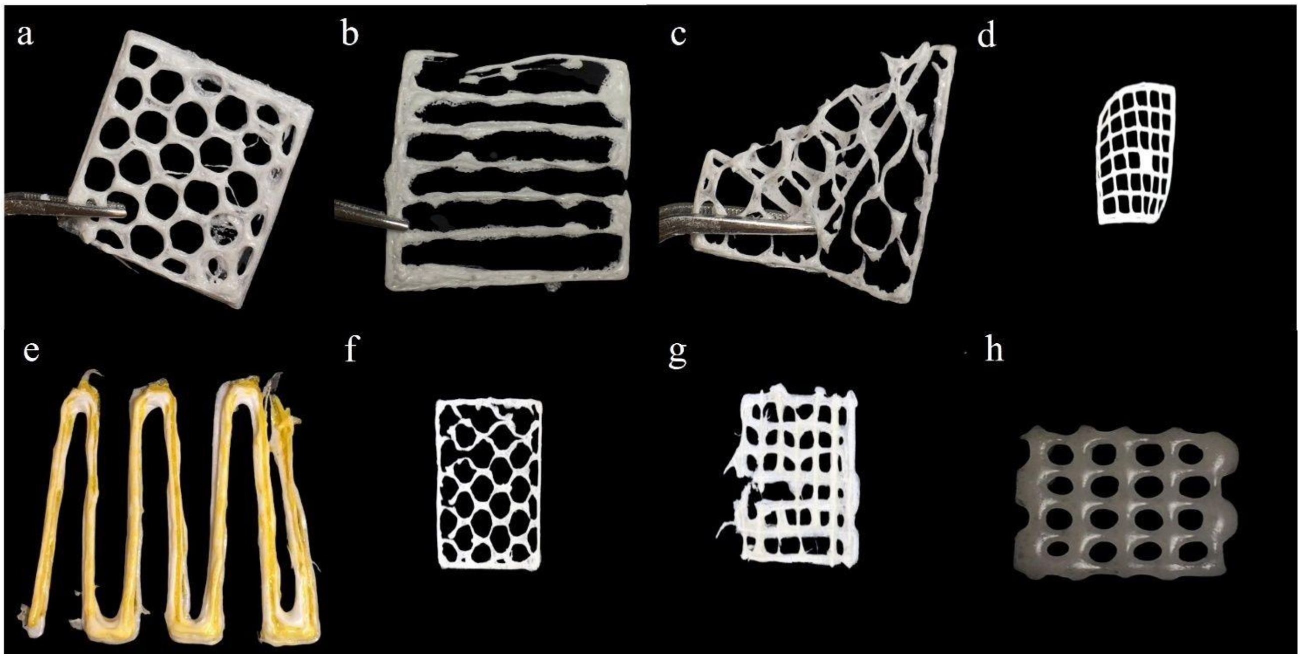

Figure S10. PCL/NaAlg meshes, a) PCL/NaAlg-2, b) PCL/NaAlg-2, c) PCL/NaAlg-1, d) PCL/NaAlg-5, e) PCL/NaAlg-4, the yellow color is due to the use of Pluronic F-127 as a core, f) PCL/NaAlg-1, g) PCL/NaAlg-3, and h) PCL/NaAlg-3

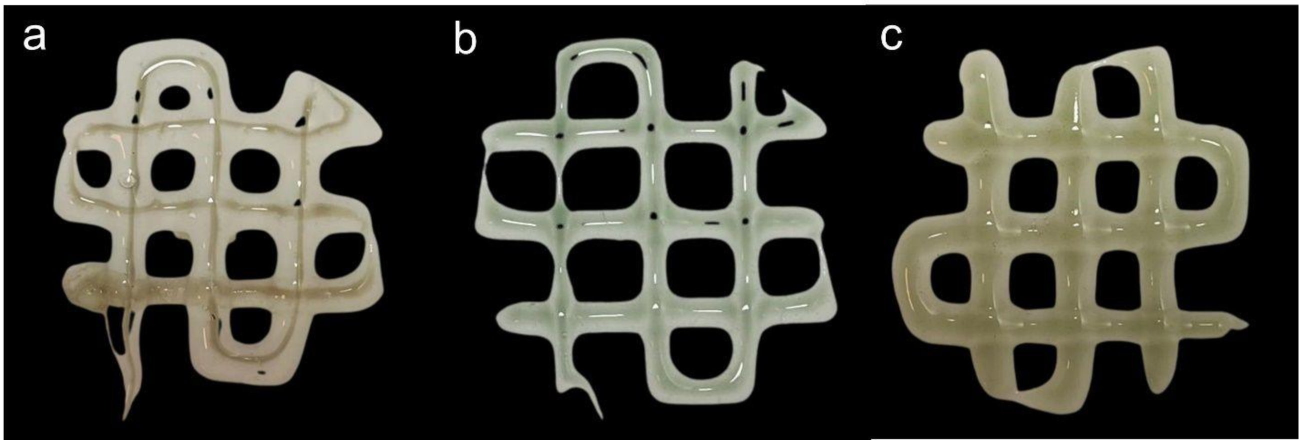

Figure S11. C-dots encapsulated in a) PEG/HPC-2, b) PEG-2, and c) PEG/HPC-1 with PCL/NaAlg shell

Confocal microscopy images

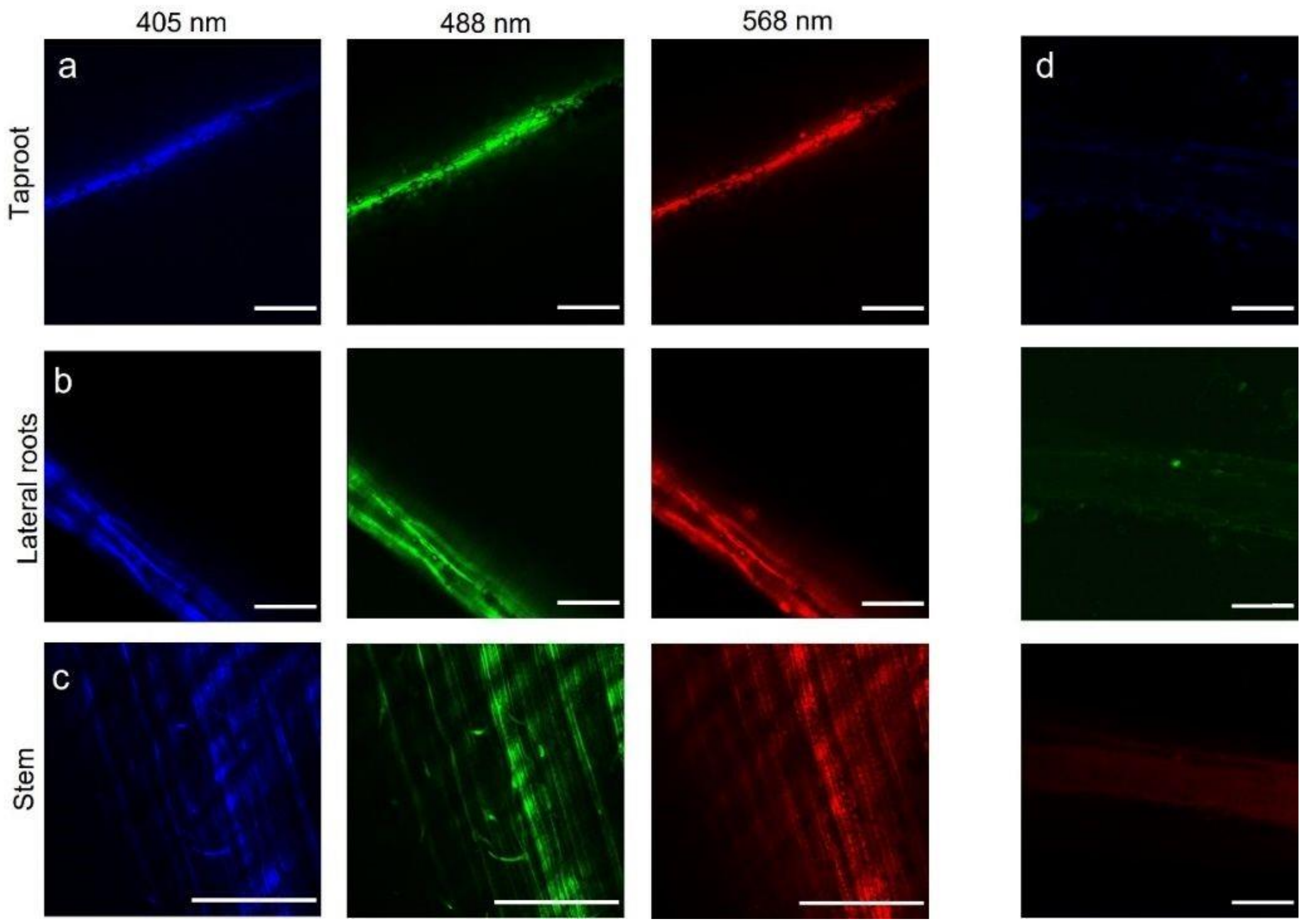

Figure S12. Row a, b, and c are the plant treated with C-dots/HPC mesh, and column d is the control plant (no treatment). The scale bar is 50 μm for a, b, d, and 200 μm for c.

Degradation of PCL/NaAlg in real soil

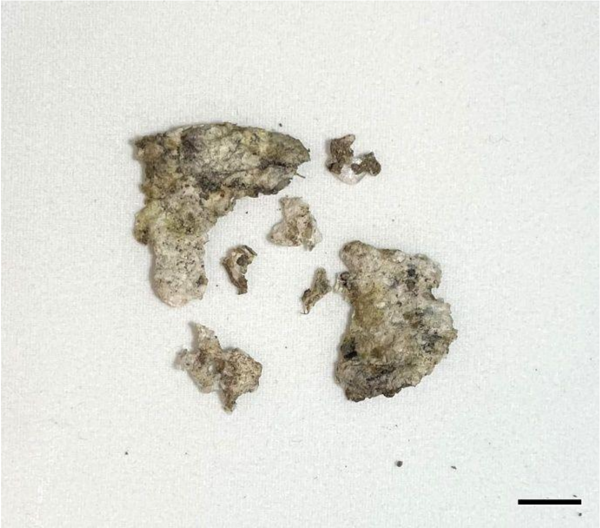

Figure S13. The degradation of PCL/NaAlg from week one to four (fixed temperature of 21°C with 79 % humidity), corresponding to ~45 wt % change, the scale bar is 5 mm.

Absorption spectra of chlorophylls and emission spectrum of C-dots

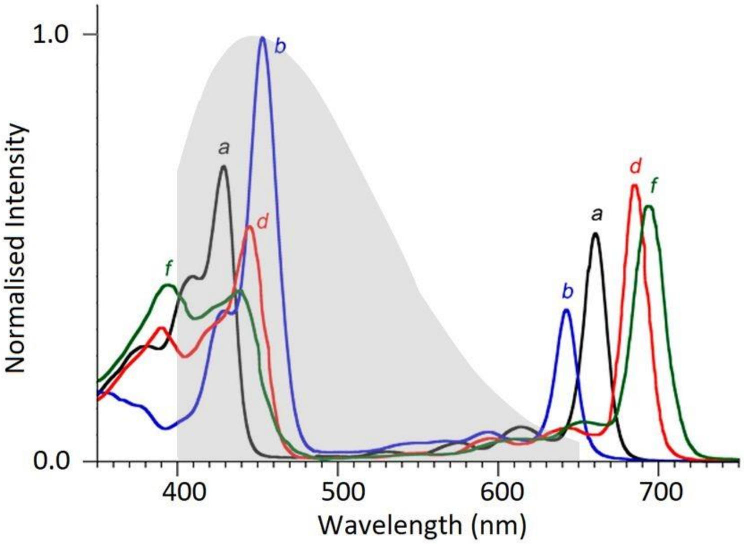

Figure S14. Absorption spectra of chlorophylls a (black), chlorophylls b (blue), chlorophylls d (red), and chlorophylls f (green) in diethyl ether at room temperature. Reproduced with permission [3]. Copyright {2015} American Chemical Society, and the threshold emission peak between 400 – 650 nm of C-dots released from the shell matrix (grey area).

Table S4. Statistical analysis of plant growth (size, N= 3)

	C-dots-HPC	C-dots-HPC-PEG	Ct-1	Ct-2	Ct-3	Ct-4	Ct-5	Ct-6
Mean (cm)	13.40	11.43	6.47	8.06	8.83	6.30	6.90	8.05
Standard deviation of the mean	0.30	2.57	0.37	1.01	1.16	0.05	0.55	0.05
Standard Deviation	0.52	4.46	0.64	1.76	2.02	0.10	0.95	0.07

References

(1) Persenaire, O.; Alexandre, M.; Degée, P.; Dubois, P. Mechanisms and Kinetics of Thermal Degradation of Poly(ε-Caprolactone). *Biomacromolecules* **2001**, *2* (1), 288–294. https://doi.org/10.1021/bm0056310.

(2) Su, T. T.; Jiang, H.; Gong, H. Thermal Stabilities and the Thermal Degradation Kinetics of Poly(ε-Caprolactone). *Polym. - Plast. Technol. Eng.* **2008**, *47* (4), 398–403. https://doi.org/10.1080/03602550801897695.

(3) Lindsey, J. S. De Novo Synthesis of Gem-Dialkyl Chlorophyll Analogues for Probing and Emulating Our Green World. *Chem. Rev.* **2015**, *115* (13), 6534–6620. https://doi.org/10.1021/acs.chemrev.5b00065.
